# Comparison of Resorbable and Non-Resorbable Osteosynthesis Material in Hallux Surgery: A Systematic Review

**DOI:** 10.3390/life13102018

**Published:** 2023-10-05

**Authors:** Manuel Coheña-Jiménez, Raquel Prieto-Domínguez, Ana Juana Pérez-Belloso, Juan Manuel Muriel-Sánchez, Álvaro Gómez-Carrión, Pedro Montaño-Jiménez

**Affiliations:** 1 Podiatry Department, University of Seville, 41009 Seville, Spain; mcohena@us.es (M.C.-J.); raquel-pd@hotmail.com (R.P.-D.); pmj@us.es (P.M.-J.); 2 Independent Researcher, Clínica Centro Lepe, Calle Rincona, 31, Lepe, 21440 Huelva, Spain; murielsanchezjm@gmail.com; 3 Independent Researcher, University Complutense of Madrid, 28040 Madrid, Spain; alvaroalcore@hotmail.com

**Keywords:** surgery, hallux valgus, resorbable, non-resorbable, osteosynthesis material

## Abstract

There are various pathologies that involve the hallux, among which hallux valgus is the most common. When conservative treatment fails, it is necessary to resort to a surgical approach. The fixation elements for osteotomies in the hallux are usually composed of metallic materials; however, today, there are numerous resorbable materials that offer numerous advantages over conventional materials. In this article, the objective is to analyze the scientific evidence through the systematic analysis of the existing literature in relation to the effectiveness of resorbable versus non-resorbable osteosynthesis material in the surgical correction of hallux deformities and compare the complications as well as the patient satisfaction and quality of life between both fixation methods. A systematic review of the literature available in the PubMed, EMBASE, Web of Science and Scopus databases and 10 studies were included. The documents were chosen following the eligibility and exclusion criteria, including experimental and observational studies evaluated with the Jadad and Newcastle-Ottawa methodological quality scale, respectively. Data were extracted from valid studies for the review, and the variables functionality, pain, angular corrections, complications, satisfaction and quality of life were observed. In conclusion, there is limited scientific evidence regarding the effectiveness of resorbable versus non-resorbable osteosynthesis material in the surgical correction of hallux deformities. All observed variables are similar regardless of the surgical technique and osteosynthesis material used.

## 1. Introduction

There are various pathologies that involve the hallux, among which hallux valgus is the most common forefoot problem in adults [1,2,3,4]. The management of this pathology generally begins with conservative treatment, but when this fails and the deformity is painful, affecting the patient’s lifestyle, it is necessary to resort to surgical treatment [5,6,7,8].

Surgical corrections of the hallux valgus are mostly osteotomies, which must be fixed with osteosynthesis materials such as needles and screws. Stainless steel and titanium are the most commonly used materials to produce these fixation devices, showing good mechanical resistance, biocompatibility, and corrosion resistance [2,3,9]. However, in recent years, the use of resorbable implants has been gaining popularity due to some advantages over conventional metal implants; since they have an elastic modulus more similar to that of bone, it is not necessary to extract them later, and they do not interfere with the post-surgical imaging tests [10,11,12,13].

Numerous resorbable fixation materials have already been used in foot surgery, such as PLA polymers, polyglycolic acid and their copolymers. However, although successful results have been reported with these materials, complications such as granuloma formation or foreign body reaction have also been reported [1,2,3,4,5,6,7,8,9,10,11,12,13,14,15,16]. Recently, resorbable magnesium screws have been introduced, which is a material that is naturally present in the human body [1,2,3,4,5,6,7,8,9,10,11,12,13,14,15,16,17,18,19,20].

The development of resorbable materials is booming and, therefore, it is necessary to know what the advantages and disadvantages of these are compared to non-resorbable materials. As there is very little scientific evidence regarding this topic, this study aims to provide knowledge about resorbable and non-resorbable materials, establishing a comparison between both in order to know their differences and indications.

## 2. Materials and Methods

The present systematic review has been designed following the recommendations of the PRISMA Declaration (Preferred Reporting Items for Systematic Reviews and Meta-Analyses) [21,22] and, in addition, the protocol has been registered in the International Prospective Registry of Systematic Reviews (PROSPERO: CRD42023431784).

### 2.1. Inclusion Criteria

The literature was selected respecting the criteria detailed below: studies conducted in adult patients with hallux deformities requiring surgical treatment were considered eligible for analysis; that the surgical correction uses resorbable and non-resorbable osteosynthesis material; that variables related to the clinical and/or radiological sphere are measured; experimental or observational studies; documents written in Spanish or English; and publication date from 2013 to the present.

Documents that consist of systematic reviews or meta-analyses and studies that do not compare the experimental group with a control group are excluded, since we want the data obtained to be as homogeneous as possible to be able to analyze and compare them later. We also excluded all the studies involving animals. Outcome measures extracted from the studies were functionality, angular corrections, pain, complications, quality of life, and patient satisfaction.

### 2.2. Databases and Search Strategy

A systematic search of the literature was carried out in the electronic databases PubMed, EMBASE, Web of Science and Scopus during the months of April–June 2023. In order to obtain the data related to the most recent and updated treatments, we decided to select articles published between 2013 and 2023.

When establishing the design for a search strategy, the first step was to formulate a structured question. To structure it, the PICO model (patient/population/problem, intervention, comparison/control, outcome) was used: P: Adult patients who present deformities in the hallux and require surgical treatment; I: Surgical correction through open surgery or minimally invasive surgery (MIS) with resorbable osteosynthesis material; C: Surgical correction by open surgery or MIS with non-resorbable osteosynthesis material; O: Effectiveness of surgery through post-surgical outcome variables related to the clinical sphere (functionality, pain level, complications, satisfaction and quality of life) and radiological sphere (angular corrections). Thus, our research question is the following: Is surgical correction of hallux deformities with resorbable osteosynthesis material effective compared to non-resorbable osteosynthesis material?

The following search strategy was implemented in the different databases: (Wire* OR “K-wire*” OR “Kirschnerwire*” OR Screw* OR Pin* OR Implant* OR “intramedullary pin*” OR “intramedullarywire*” OR fixation) AND (Resorbable OR Bioresorbable OR Absorbable OR Bioabsorbable OR Degradable OR Biodegradable OR “Absorbablefixation” OR “Resorbablefixation” OR “Poly-L-lactideacid” OR Copolymer* OR PLLA OR PLDLA OR PLA OR PGA OR Polylactate OR “Polyglycolide” OR “Polylacticacid” OR “Polylactideacid” OR “polyglycolicacid” OR Magnesium) AND (Arthrodesis OR osteotomy OR osteotomies OR correction OR “halluxsurgery” OR “Interphalangealjoint” OR bunionectomy OR “forefootsurgery” OR “digital surgery”) AND (DIP OR PIP OR Forefoot OR Digital OR hallux OR “halluxvalgus” OR “halluxabductusvalgus” OR “halluxvarus ” OR “firstray” OR bunion OR “Interphalangealjoint” OR digit* OR “toe joint”).

Two independent reviewers (R.P.-D. and J.M.M.-S.) assisted with conducting and validating the research. Only articles written in English and Spanish were accepted.

### 2.3. Selection of Studies

**Figure 1:**
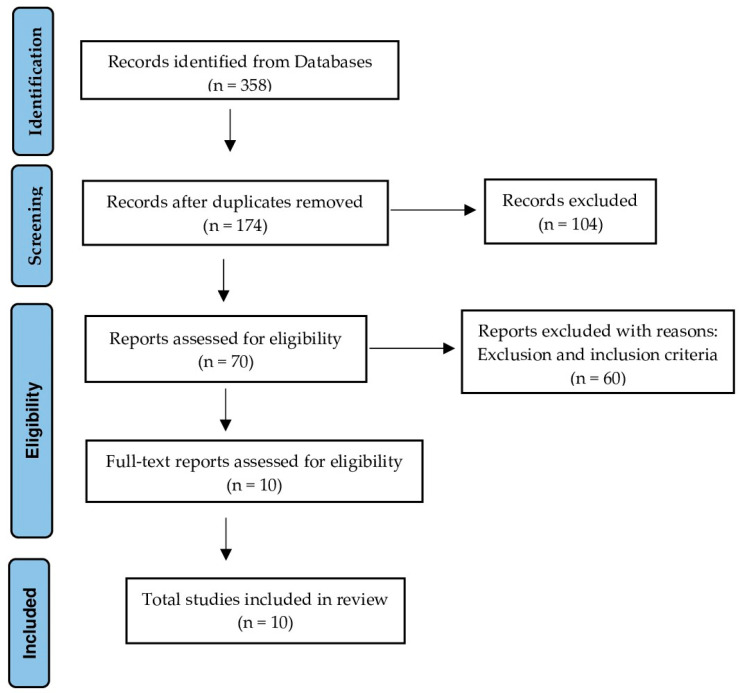
Flow chart.

The articles that emerged from the research were independently reviewed by two independent reviewers (R.P.-D. and J.M.M.-S.). Once the total number of documents was obtained from the EMBASE, PubMed, Web of Science and Scopus databases and having applied the temporal filter from 2013 to 2023, the first step of this process consisted of locating the duplicate studies in order to discard them manually. Subsequently, the titles and abstracts of the studies were analyzed to evaluate their eligibility using the established inclusion and exclusion criteria, screening those that did not meet these criteria. Those that were not in full text and systematic reviews with or without meta-analysis were also excluded. Finally, the full text of those studies that were susceptible to selection was reviewed and included in the present systematic review. Disagreements were resolved through group discussion with arbitration by the senior author. This entire process is recorded in the flow chart (Figure 1).

### 2.4. Data Extraction

To follow this process, a standardized table was created where the characteristics that we considered most relevant for each study were added: author and year of publication, type of study, study population, sample size, intervention performed, follow-up, variables of interest, main results, conclusions and limitations. Due to the heterogeneity of the clinical studies and the population sample analyzed in the different studies, some data were missing or cannot be extrapolated; therefore, missing data have been considered in the presentation of our results.

### 2.5. Quality Assessment

The quality of clinical trials was evaluated using the Jadad scale [23], and the quality of the case-control studies was assessed using the Newcastle-Ottawa scale [24,25]. The Jadad scale allows evaluating the methodological quality of clinical trials and has 5 items with the maximum score of the scale being 5 points. If the score obtained is less than 3 points, the clinical trial is considered weak [23]. The Newcastle-Ottawa scale is based on a star system through which different studies are evaluated based on 3 categories, which contain a certain number of items. Studies that have earned a score of 7 to 9 stars are classified as high quality. Those with a score of 4 to 6 stars are considered studies at high risk of bias, and those with scores of 0 to 3 stars have a very high risk of bias [24,26].

## 3. Results

After carrying out the search in the different databases with the aforementioned strategy and adding the temporal filter from 2013 to 2023, a total of 358 documents have been obtained. Of this total, 184 studies have been eliminated due to being duplicates, leaving 174 documents. After reading the title and abstract, 104 studies were eliminated since they were interventions other than those proposed in our objectives, leaving us with 70 documents. Of these, 60 were excluded after reading the full text according to the inclusion and exclusion criteria, finally leaving us with a total of 10 articles.

We were therefore left with a total of 10 studies for the final analysis (Figure 1).

### 3.1. Quality of Studies

According to the Jadad scale, clinical trials conducted by Plaass et al. [27] and Windhagen et al. [28] have obtained a score equal to 3; therefore, they are considered clinical trials with adequate methodological quality. According to the Newcastle-Ottawa scale, eight case-control studies have obtained a score greater than 7 and thus are considered to be of high quality.

### 3.2. Materials

Of the 10 documents included in the present review, with respect to materials, six studies compare magnesium screws with titanium screws, two studies compare polylactic acid (PLA) needles with Kirschner needles, one study compares L-lactide and trimethylcarbonate copolymer needles with titanium screws, and finally, one study compares poly L-lactic acid (PLLA) and poly D, L-lactic acid (PDLLA) copolymer needles with titanium screws.

### 3.3. Functionality

Of the 10 studies, nine measure functionality using various scales: seven studies use the American Foot and Ankle Society Hallux Metatarsophalangeal-Interphalangeal Score (AOFAS-MTP-IP), three studies measure the range of motion of the 1st metatarsophalangeal joint (ROM 1st AMTF) using a goniometer, two studies use the Manchester-Oxford Foot Questionnaire (MOXFQ), one study includes the Foot Function Index (FFI) and another study uses the Foot and Ankle Outcome Score (FAOS). Furthermore, one study measures functionality by assessing functional impairment using the visual analogue scale (VAS), and another study does so by assessing the patients’ ability to walk using a nominal scale.

### 3.4. Angular Corrections

Of the 10 studies, seven articles measure angular corrections: In seven studies, the hallux valgus angle (HVA) and the intermetatarsal angle (IMA) are measured, in two studies, the distal metatarsal joint angle (DMAA) is measured, and the authors measure the hallux interphalangeal angle (HIA) in only one study.

### 3.5. Pain

Of the 10 studies, eight measured the pain of patients: five articles used the VAS and one article used the Numerical Rating Scale (NRS). Furthermore, in one study, the pain of the first metatarsophalangeal joint (1st AMTF) perceived by the patient in his daily life and during the follow-up examination was evaluated using a nominal scale.

### 3.6. Complications

Of 10 studies, nine compile the complications that have appeared during the follow-up period.

### 3.7. Quality of Life and Satisfaction

Of the 10 studies, two measure the quality of life perceived by patients, and three studies measure their satisfaction after the procedure. To measure quality of life, different scales have been used: two studies use the Short-Form Health Survey (SF-36 health questionnaire), while one study uses the Foot and Ankle Ability Measure (FAAM) referring to activities of daily living (ADL) and sports (SPORT). Finally, one study uses the EuroQoL 5-Dimension 3-Level (EQ-5D-3L). To measure satisfaction, one study has used the Coughlin scale and the Likert scale.

The summarized data in our systematic review are reported in Table 1.

## 4. Discussion

The main objective of this systematic review is to review the current scientific evidence through the systematic analysis of the existing literature in relation to the effectiveness of resorbable versus non-resorbable osteosynthesis material in the surgical correction of hallux deformities to improve functionality, pain and angular corrections as well as compare the associated complications and the satisfaction and quality of life perceived by patients. A total of 10 studies have been included that compare various types of resorbable osteosynthesis material such as magnesium screws and polydioxanone, polylactic acid, L-lactide-trimethylcarbonate and PLLA-PDLLA needles with non-resorbable osteosynthesis material such as Kirschner wires and titanium screws.

Based on the objectives set and the variables selected, we will proceed to discuss the findings found among the different studies analyzed.

### 4.1. Functionality

All studies that have assessed functionality using the AOFAS-MTP-IP scale before and after surgery have agreed that there was a significant improvement in the score at the end of follow-up in comparison with pre-surgical values both in the group where resorbable material was used and in the group where non-resorbable material was used [37,38,39]. In addition, the post-surgical AOFAS-MTP-IP scale values did not show significant differences between the study groups. However, the study by Wendelstein et al. [30] does not offer us the pre-surgical values of the scale, therefore, it cannot be known if there was an improvement in the score at the end of the follow-up. However, the final pre-surgical values that we can observe in these studies are good with no significant difference between the different groups.

Regarding the ROM 1st AMTF, in the study by Windhagen et al. [28], there was an increase in this after surgery compared to pre-surgical values in the group where magnesium screws were used and in the group where titanium screws were used. In contrast to the results obtained in previous studies, Plaass et al. [27] and Acar et al. [35] observed a significant decrease in ROM 1st AMTF in their studies regardless of whether the patient had a magnesium screw or a titanium screw. What all the studies do have in common is that no significant difference was found between the study groups regarding the final ROM 1st AMTF values.

Song et al. [29] analyzed functionality using MOXFQ in their study and observed that there was a significant improvement in scores in all domains after surgery compared to the score obtained before surgery. This result was obtained in both study groups, and they did not show significant differences in post-surgical scores. A similar result was obtained by Atkinson et al. [32] in their study, since all the parameters of the questionnaire improved significantly after surgery, with the parameters “foot pain” and “social interaction” being the ones that showed better improvement levels in both groups. In the final results, there was no significant difference between the groups except in the parameters “walking standing” and “index”, being significantly better in the group where the osteotomy was fixed with magnesium screws.

Wendelstein et al. [30] wanted to evaluate the FFI at the end of the follow-up and were able to observe that there were no significant differences in the values obtained between the three study groups; however, Kirschner needles were the ones that obtained the best FFI followed by the magnesium screws and finally the titanium screws.

Only Atkinson et al. [32] used the FAOS in their study. In it, they noticed that the score improved significantly in all patients in both groups with no significant differences between them for any of the individual scoring parameters.

Finally, in the study by Wendelstein et al. [30], the VAS was used to assess functional deterioration after surgery, and the results showed no significant differences between the three types of groups with a very low score in the VAS.

### 4.2. Angular Corrections

Regarding the HVA and the IMA, all the studies that measure these angles stated that thanks to the surgery, there had been a significant decrease in them at the end of the study follow-up compared to the pre-surgical results both in the group where resorbable material is used and in the group where non-resorbable material is used.

Furthermore, in most of the articles, there were no significant differences between the study groups regarding the pre-surgical and post-surgical angular values; therefore, the degrees of surgical correction were similar. However, in the study by Choo et al. [31], there were more degrees of correction of the HVA and IMA in the group where titanium screws were used than in the group where magnesium screws were used. On the contrary, the results obtained by Wendelstein et al. [30] showed significantly higher degrees of correction of the IMA in the group where magnesium screws were used compared to metallic Kirschner wires. This same result was obtained in the study by Morandi et al. [36], where the degrees of IMA correction were also significantly higher in the resorbable group, where PLLA and PDLLA needles were used, compared with titanium screws.

Only three articles have taken into account angles other than HVA and IMA. In the studies by Windhagen et al. [28] and Komur et al. [34], the DMAA was included, and the result was a significant decrease in both studies after surgery in both the group where resorbable material was used and in the group where non-resorbable material was used. Finally, Song et al. [29] decided to include the HIA in their study, and it was concluded that there was also a significant decrease in the angle after surgical correction in both the resorbable PLA needles group and the non-absorbable group of Kirschner needles.

### 4.3. Pain

The studies conducted by Choo et al. [31], Windhagen et al. [28], and Komur et al. [34] have used the VAS to evaluate the pain of patients, and all have concluded that there has been a significant decrease in pain at the end of follow-up compared to the level of pain before surgery both in the group where resorbable material was used and in the group where non-resorbable material was used.

These results agree with the study by Acar et al. [35]; however, in this study, a patient who underwent surgery with a titanium screw had to have the implant removed after six months due to the pain he suffered in his daily activities. In the study by Plaass et al. [27], assessed pain using the NRS and also found a significant decrease in pain three years after surgery in both groups. No patient who underwent surgery with magnesium screws had residual pain; however, in three patients with titanium screws, the pain persisted: two of them felt slight pain when running and the other patient felt pain when running, during walking and in repose.

The study by Wendelstein et al. [30] has not assessed pain before surgery; therefore, a comparison between pre-surgical and post-surgical values cannot be established. However, the post-surgical pain values on the VAS scale were quite low in both groups and in both studies.

All the studies mentioned above agree that there is no significant difference in post-surgical pain values between patients in the resorbable group and patients in the non-resorbable group.

### 4.4. Complications

Due to the heterogeneity of complications, the discussion of data between the different studies included in the review becomes complex.

The only study where no intraoperative or postoperative complications were observed in both groups was that of Atkinson et al. [32]. Regarding implant removal due to discomfort, no resorbable implant had to be removed in any of the studies. This did not happen with non-resorbable implants: in the studies by Plaass et al. [27], Windhagen et al. [28], Choo et al. [31], and Acar et al. [35], the titanium screw had to be removed in one patient due to the discomfort it caused.

The studies carried out by Plaass et al. [27] and Song et al. [29] showed no significant differences between post-surgical complications between the study groups. Similar results were found in the remaining studies where complications did not differ between groups. Acar et al. [35] also did not observe a significant difference between both groups in complications except for the accumulation of gas in the tissues surrounding the osteotomy, since this complication occurred in a large number of patients, all with magnesium screws. Like the previous study, Klauser [33] also found no significant differences between the groups in most complications; however, in 40% of the patients who had magnesium screws, radiological findings appeared such as osteolysis, areas lytics, radiolucency and demineralization around the screw. Both the accumulation of gas in the surrounding tissue and the radiological findings mentioned above are the result of the magnesium degradation process, being temporary and not intervening with the consolidation of the osteotomy.

### 4.5. Quality of Life

In the study carried out by Atkinson et al. [32], the quality of life perceived by the patients was evaluated using the EQ-5D-3L before and after surgery, resulting in a significant improvement in the quality of life of the patients after undergoing surgery—both in the group where fixation was performed with magnesium screws and in the group where titanium screws were used. Similar results were obtained by Choo et al. [31] using the SF-36 health questionnaire. In the group where magnesium screws were used, there was a significant improvement in all domains of the questionnaire except in the “emotional role” domain, which also improved, although not significantly. In the group where titanium screws were used, there was a significant improvement in all domains of the questionnaire.

Plaass et al. [27] evaluated the quality of life of the patients only after surgery using the SF-36 health questionnaire and the FAAM. A comparison of pre-surgical and post-surgical values could not be made; however, the quality of life of the patients after surgery was good, and there were no significant differences between the resorbable and non-resorbable group. This result was the same for the study by Atkinson et al. [32]; however, in the study by Choo et al. [31], the “general health” domain of the SF-36 health questionnaire was significantly better in the group where magnesium screws were used.

### 4.6. Satisfaction

Regarding patient satisfaction based on the procedure and result, Plaass et al. [27] were able to conclude that all patients in the study were very satisfied with the surgery, would undergo it again if necessary and would recommend it. Similar results were obtained by Windhagen et al. [28] in their study, since all patients were very satisfied with the surgery except one belonging to the resorbable group who had healing problems and was dissatisfied with the procedure.

Song et al. [29] assessed patient satisfaction using the Coughlin scale and the Likert scale, also being able to observe excellent levels of satisfaction. Although the differences were not significant between the study groups, the level of satisfaction was slightly higher in the patients who underwent surgery with PLA needles. We found the same thing in the study by Wendelstein et al. [30] where the patients were also asked if they would undergo the same intervention again, finding a significant difference since the resorbable material group had greater satisfaction.

## 5. Conclusions

Based on the objectives set and the results obtained in this systematic review, the following can be concluded. First, there is limited scientific evidence regarding the effectiveness of resorbable versus non-resorbable osteosynthesis material in the surgical correction of hallux deformities to improve functionality, pain and angular corrections. All existing studies show improved functionality, decreased pain and effective angular corrections after surgery regardless of the surgical technique and osteosynthesis material used. Second, regarding post-surgical complications, implant removal has only occurred in patients whose surgery used non-resorbable osteosynthesis material. The rest of the complications are quite similar regardless of the osteosynthesis material used. Finally, regarding patient satisfaction and the quality of life perceived by them after surgery, both resorbable and non-resorbable osteosynthesis material improve these variables with resorbable osteosynthesis material offering somewhat better results.

Regarding the limitations of this systematic review, it is worth highlighting the small number of studies that currently exist on the selected topic, most of them being observational case-control studies. This type of study has a lower methodological quality and scientific evidence than that provided by clinical trials; therefore, we have decided to include the few clinical trials that we have found, although this increases the heterogeneity of the studies selected for this review. However, these clinical trials are of medium-low quality. Another limitation to highlight is that there is a wide variety of resorbable hardware that differs in materials, shape, and resorption time. This represents a bias because, considering the low volume of literature, some of the hardware has been evaluated in very few studies. Due to all of the above, the conclusions obtained in this review will be limited to the articles that currently exist. Therefore, it would be advisable to carry out future lines of research where good-quality randomized clinical trials are carried out in order to increase scientific evidence on the topic of study.

## Figures and Tables

**Table 1:** Studies included in the review and main features: results and conclusions.

Author and Year	Type of Study	Sample Size	Middle Ages	Intervention/ Follow-up	Main Features/Results	Conclusions
Plaass et al. (2018) [27]	Randomized clinical trial	EG: 8 patients CG: 6 patients	EG: 56 ± 8.9 years CG: 52 ± 9.0 years	EG: Chevron + MAGNEZIX^®^ Magnesium Screw CG: Chevron + Titanium Screw /36 months	ROM 1º AMTF: No significant difference between groups in pre vs. post-surgical results. Significant reduction in ROM in both groups at 3 years. AOFAS-MTP-IP; Pain (NRS): No significant difference between groups in pre- vs. post-surgical results. Significant improvement in both groups at 3 years. FAAM ADL; FAAM Sport; SF-36: No significant differences between groups. All patients were very satisfied with the surgery. Associated complications: Screw extraction in 1 patient of the CG.	No significant differences between groups. Magnesium screws show comparable results to titanium screws.
Winha-gen et al. (2013) [28]	Randomized clinical trial	EG: 13 patients CG: 13 patients	EG: 57.2 years CG: 49.9 years	GE: Chevron + MAGNEZIX^®^ Magnesium Screw GC: Chevron + Titanium Screw /6 months	ROM 1st AMTF, AOFAS-MTP-IP; Pain (VAS): No significant difference between groups at the follow-up visit. Improvement at the end of follow-up without observing stiffness of the 1st TMA, Pain (VAS). IMA, HVA and DMAA: Decrease in angles in both groups at the end of the follow-up. 23/24 patients were very satisfied Complications: Screw extraction: 1 CG patient. Post-surgical discomfort: EG: 2 patients and CG 1 patient. Delayed consolidation: EG: 2 patients, CG: 1 patient.	No significant differences between groups. Magnesium screws show comparable results to titanium screws. Complications were considered as a result of the surgical procedure itself.
Song et al. (2021) [29]	Cases and controls study	EG: 33 patients CG: 28 patients	EG: 58 years (21–77) CG: 56 years (22–75)	GE: Chevron + PLA needle trim-it ^®^ GC: Chevron + Kirschner needle /36 months	No significant difference between groups at 3 years of follow-up. MOXFQ: Significant improvement at 3-year follow-up compared to pre-surgical values in the EG. Patient satisfaction: EG: Coughlin scale: 47.6% excellent; 40.5% good; 7.1% medium; 4.8% little. Likert scale: Mean satisfaction: 3.2 (range: 1–4). IMA, HVA, HIA: In the EG, it improved significantly after 3 years of follow-up compared to pre-surgical values. Complications: EG: 7/42 (16.6%)/12/38 (31.5%) feet had complications.	Fixation with PLA needles obtained favorable clinical and radiological results with fewer complications and providing stable fixation until bone consolidation.
Wen-delstein et al. (2021) [30]	Cases and controls study	GE: 16 feet GC1: 16 feet GC2: 16 feet	GE: 60.6 ± 12.1 years GC1: 60.2 ± 11.5 years GC2: 59.1 ± 11.3 years	GE: Chevron + MAGNEZIX^®^ magnesium screw GC1: Chevron + Autofix ^®^ titanium screw GC2: Chevron + Kirschner needle /12 months	AOFAS-MTP-IP; FFI: No significant difference between the 3 groups in the post-surgical follow-up results. IMA and HVA: Significantly improved at 12-month follow-up compared to pre-surgical values in the 3 study groups. IMA: Significantly smaller post-surgical angles in the GE compared to the K-wire group. However, between the GE and the titanium screw group, there were no statistically significant results. HVA, Pain, Satisfaction: No significant difference between the 3 groups in the postoperative results at follow-up. Satisfaction: All GE patients would undergo surgery again if necessary, resulting in significantly higher satisfaction in this group. Complications: GE: Tenderness in the osteotomy area (2.13%); Implant rupture (2.13%); Dislocation of the metatarsal head (2.13%); Early radiolucency around the implant (3.19%); GC1: Deep infection (1.6%); Superficial infection (1.6%); Delayed bone healing (1.6%); Tenderness in the osteotomy area (2;13%); Early radiolucency around the implant (1.6%). GC2: Deep infection (1.6%); Bunion recurrence (1.6%); Tenderness in the osteotomy area (1.6%); 15 of 16 patients underwent elective K-wire removal.	Screws: Show results comparable to titanium screws. Patients showed a higher satisfaction rate and were significantly more likely to repeat the same procedure. The IMA was lower compared to the K-wire group, as the additional compression properties of the magnesium screw cause more stable osteosynthesis and lower intermetatarsal angles.
Choo et al. (2019) [31]	Cases and controls study	GE: 24 patients CG: 69 patients	GE: 54.5 ± 12.0 years GC: Non indicated	GE: Scarf + magnesium screwsMAGNEZIX ^®^ GC: Scarf + titanium screws. /12 months.	AOFAS-MTP-IP: No significant difference between groups in post-surgical results. Significant improvement in both groups one year after surgery. Pain (VAS): Significant improvement in both groups one year after surgery. SF-36: GE: Significant improvement in all domains of the questionnaire except role limitation due to emotional problems. SF-36: GC: Significant improvement in all domains of the questionnaire. The “general health” domain was significantly better in the GE than in the GC. HVA; IMA: Significant decrease in both groups in post-surgical results compared to pre-surgical results. There is a significant difference between groups in the means of post-surgical results, being significantly better in the GC than in the GE. Complications: GE: Superficial cellulitis (12.5%) and localized post-surgical neuropathic pain (4.2%). GC: Superficial cellulite (4.3%); complex regional pain syndrome (1.4%) and implant removal due to discomfort (1.4%).	Magnesium screws show comparable results to titanium screws. There were no significant differences between groups in functional outcomes, although radiological improvements were significantly better in the GC.
Atkinson et al. (2019) [32]	Cases and controls study	GE: 11 patients GC: 25 patients	GE: 38 (25–51) years. GC: 41 (26–72) years.	GE: short Scarf + MAGNEZIX^®^ magnesium screw GC: Yeshort scarf + titanium screw /12 months	MOXFQ: All scoring parameters improved significantly after surgery (GE and GC). The highest levels of improvement were with the parameters “foot pain” and “social interaction”. However, the GE had a significantly greater improvement in the parameters “walk/stand” and “index”. FAOS; EQ-5D-3L: All scoring parameters improved significantly after surgery in both groups. There were no significant differences when comparing post-surgical scores between the two groups for any of the individual scoring parameters. Complications: No intra or post-operative complications were observed in both groups. No patient in either group required surgery to remove the implant.	Screws are clinically effective and safe, showing results comparable to titanium screws. The material characteristics of magnesium screws are different from those of conventional metal screws, requiring a learning process to be able to use them correctly.
Klauser (2019) [33]	Cases and controls study	GE: 100 patients CG: 100 patients	GE: 50.9 years GC: 52.3 years	GE: Chevron or Youngswick -Austin + MAGNEZIX^®^ magnesium screw GC: Chevron or Youngswick -Austin + Fixos ^®^ titanium screw /3 months	Complications: No significant difference between groups. Delay in healing: 3 patients in GE and 4 patients in GC; Soft tissue irritation due to the implant: No patients in the GE and 1 patient in the GC; Site infection: 2 patients in GE and 1 patient in GC; Screw breakage: 1 patient in the GE and none in GC. Radiological findings: GE: Correct placement of the implants and bone healing without anomalies (60% of cases). Phenomena such as osteolysis, lytic areas, radiolucency or demineralization were found around the magnesium screw (40% of cases). GC: Correct placement of the implants and early signs of bone consolidation and healing without anomalies (100% of cases).	Magnesium screws show comparable results to titanium screws. The radiological phenomena found in patients with magnesium screws are associated with their degradation process, subsequently disappearing.
Komur et al. (2018) [34]	Cases and controls study	GE: 40 patients CG: 40 patients	GE: 43.1 years GC: 43.5 years	GE: Chevron + resorbable L- lactide and trimethylcarbonate OTPS^®^ copolymer needle. GC: Chevron + titanium screw /3.5 months	AOFAS-MTP-IP: No significant difference between groups in postoperative results. The score improved significantly after surgery in both groups. Pain (VAS): No significant difference between groups in postoperative results. Significant improvement in both groups. IMA; HVA; DMAA: No significant difference between groups in postoperative results. Significant angle decrease in both groups.	Both fixation methods are safe and reliable for the surgical correction of the hallux valgus under appropriate conditions and when performed by an experienced surgeon; however, the cost of the resorbable material is higher.
Acar et al. (2018) [35]	Cases and controls study	GE: 16 patients GC: 15 patients	GE: 49.9 ± 15.1 years GC: 48.5 ± 14.6 years	GE: Chevron + MAGNEZIX^®^ magnesium screw GC: Chevron + Titanium Screw /GE: 19 ± 6.8 months. GC: 16 ± 6.19 months	ROM 1º AMTF, AOFAS-MTP-IP; Pain (VAS): No significant difference between groups in the final pre- and post-surgical results. Significant decrease in ROM in both groups at the end of follow-up compared to pre-surgical values. AOFAS-MTP-IP; Pain (VAS): Significant improvement in both groups. IMA; HVA: No significant difference between groups in pre-surgical, early and final post-surgical results. Significant decrease in the angle in both groups in the final results. Complications: Implant removal rate: GE: none; GC: 1 case. Statistically similar in both groups; Mild edema and hyperemia around the surgical incision: GE: 1 case; GC: none. Statistically similar in both groups. Gas accumulation in soft tissues: GE: 13 cases; CG: none.	Similar clinical and radiological results in both groups. Magnesium screws provide the advantage of a lower implant removal rate. The radiological findings of magnesium screws are the result of their degradation process; therefore, it is necessary for surgeons and radiologists to be familiar with these images for their correct interpretation.
Morandi et al. (2013) [36]	Cases and controls study	GE: 251 patients GC: 132 patients	GE: 58.6 (19–74) years GC: 63.2 (26–80) years	GE: Chevron + resorbable needle composed of a copolymer of PLLA and PDLLA Osteo-Tec^®^ GC: Chevron + Integra^®^ Bold Titanium Screw /12 months	AOFAS-MTP-IP and HVA: No significant difference between groups in the final results. Significant improvement in both groups at the final follow-up. IMA: Significant difference between groups in the final results. Significant decrease in both groups at the final follow-up. Complications: Giant cell granuloma: 0.7% (GE); Slight loss of correction: 3.2% (EG); Dorsal edema, erythema and pain in the hallux when wearing shoes: 0.6% (GC). The screw was removed after one year without loss of correction. Satisfaction: 100% of patients declared themselves very satisfied with the procedure.	Both methods are effective, allowing important angular corrections and with a low complication rate. The only difference found between the two fixation methods was the cost, since the titanium screw is 25% cheaper than the resorbable one. The selection of the patient, the implant and the surgical technique helps to minimize complications.

## Data Availability

Please contact aperez30@us.es with any data requests.
